# Combination of near-infrared and thermal imaging techniques for the remote and simultaneous measurements of breathing and heart rates under sleep situation

**DOI:** 10.1371/journal.pone.0190466

**Published:** 2018-01-05

**Authors:** Menghan Hu, Guangtao Zhai, Duo Li, Yezhao Fan, Huiyu Duan, Wenhan Zhu, Xiaokang Yang

**Affiliations:** Shanghai Institute for Advanced Communication and Data Science, Shanghai Key Laboratory of Digital Media Processing and Transmission, Shanghai Jiao Tong University, Shanghai, China; Huazhong University of Science and Technology, CHINA

## Abstract

To achieve the simultaneous and unobtrusive breathing rate (BR) and heart rate (HR) measurements during nighttime, we leverage a far-infrared imager and an infrared camera equipped with IR-Cut lens and an infrared lighting array to develop a dual-camera imaging system. A custom-built cascade face classifier, containing the conventional Adaboost model and fully convolutional network trained by 32K images, was used to detect the face region in registered infrared images. The region of interest (ROI) inclusive of mouth and nose regions was afterwards confirmed by the discriminative regression and coordinate conversions of three selected landmarks. Subsequently, a tracking algorithm based on spatio-temporal context learning was applied for following the ROI in thermal video, and the raw signal was synchronously extracted. Finally, a custom-made time-domain signal analysis approach was developed for the determinations of BR and HR. A dual-mode sleep video database, including the videos obtained under environment where illumination intensity ranged from 0 to 3 Lux, was constructed to evaluate the effectiveness of the proposed system and algorithms. In linear regression analysis, the determination coefficient (R^2^) of 0.831 had been observed for the measured BR and reference BR, and this value was 0.933 for HR measurement. In addition, the Bland-Altman plots of BR and HR demonstrated that almost all the data points located within their own 95% limits of agreement. Consequently, the overall performance of the proposed technique is acceptable for BR and HR estimations during nighttime.

## Introduction

Sleep plays an important role in people's mental and physical health as well as well-being throughout life. Sleep disorders have been considered as a significant public health issue, with nearly 30 percent of the worldwide adults suffering from them [1]. It is noteworthy that a vast number of undiagnosed people are unaccounted due to the high costs and inconvenient diagnostic methods [2]. Some chronic health problems, such as obesity and diabetes, are reported to be correlative with the sleep disorders [3]. Sleep disorders can also influence the way one person thinks, works, reacts and learns [4], and thus may result in a series of terrible accidents, such as traffic crashes. According to the American Academy of Sleep Medicine, the specific sleep disorder will appear with some unique symptoms [5]. Therefore, it is essential to gather the various sleep related information including sleep time, body movement times, sleep position, heart rate (HR) and breathing rate (BR) for the diagnosis of sleep disorders. However, most current measurement technologies are intrusive, cumbersome, inconvenient and expensive, which in turn bring about discomfort, stress and even soreness of the patients. Furthermore, to record the sleep related data, the patients have to use many on-body sensors and sleep in the strange environments. This may have an impact on the collection of natural and accurate sleep information. For the above reasons, the diagnosis of sleep disorders has become an extremely difficult task.

Optical measurement techniques offer the potential for the contactless and unobtrusive estimation of sleep related information. The Microsoft Kinect sensor, an off-the-shelf depth camera, was utilized to remotely recognize the sleep movement and posture [6]. The same device was used by the other group of investigators who extracted frequency and regularity features from breathing curves for the sleep disorders analysis [7]. The capability of custom-made infrared imaging system for sleep monitoring had been also studied. A CMOS camera coupled with an infrared light operating at wavelength of 825 nm was applied for detecting the movement pattern during sleep [8]. Martinez and Stiefelhagen captured the fixed infrared dot matrix, and subsequently, the BR was estimated from the displacements of some dots caused by chest movements [9]. Apart from the methods based on movement detection, the variations of pixel intensities are also widely used signals to be served as sleep disorder symptoms. Thermal imaging technique can detect the radiation emitted by the objects whose temperature exceeds absolute zero, and therefore, it is very suitable for executing the tasks during night such as sleep monitoring. In terms of sleep apnea event detection, the thermal imagery was employed to extract the temperature variations around the nostril regions [10].

Among the sleep related data, BR and HR are two critically important factors reflecting the quality of sleep. For example, nocturnal paroxysmal dystonia, one of the sleep disorders, will occur with or be preceded by the breathing pause and slowed HR [5]. BR is defined as the number of breaths a person takes per minute. Some literature have reported to use the optical based approaches for measuring the BR from a long distance. Nam et al., applied the built-in camera on smartphone to film the motions of chest and abdomen, and the BR was then derived with the average median error below 2% [11]. In contrast to the displacement change, temperature variation is more significant and therefore more suitable for estimating breathing signature. Basu et al., used the infrared thermography to monitor such variation for the subsequent computation of BR [12]. HR is the speed of the heartbeat which is measured by the number of heart beats per minute. Under a certain illumination, subtle color changes caused by blood circulation allow us to detect HR through the camera [13]. Yan et al., took advantage of this point to measure HR from facial RGB video illuminated by natural light [14]. Although some investigators developed the filters for eliminating the effects of illumination variations, this is still a highly illumination-dependent solution for HR measurement. For measuring these bio-signals during night, the systems combining cameras in different wavelengths can provide more accurate and robust results than those using the single sensor.

Very limited attempts have been undertaken to implement multi-camera systems for measurements of BR and HR. Gupta et al., established a synchronized multimodal imaging system containing an RGB camera, a monochrome camera and a thermal camera for estimating the HR [15]. The results of their experiments demonstrated that the imaging system inclusive of more spectral channels could achieve more accurate and robust measurement. Procházka et al., used the RGB, depth and infrared sensors to concurrently measure BR and HR under the natural environment [13]. A dual-wavelength imaging system operating at 611 nm and 880 nm was applied for blood oxygen saturation estimation [16]. The feasibility of this system in BR and HR measurements requires further studies. In conclusion, due to their properties, the above systems may be unsuitable for measurements of vital physiological signals during night.

Therefore, considering the environmental characteristics of night, we leverage an infrared camera equipped with IR-cut lens and a far-infrared imager to develop a dual-camera imaging system, thus allowing to record the infrared and thermal videos. Furthermore, an infrared light array associated with a photosensitive sensor is adopted to switch the system to the night mode, in which the system can simultaneously obtain infrared and thermal image sequences.

The main contributions of this paper are to present: (1) a dual-camera setup that can carry out the simultaneous and unobtrusive BR and HR measurements during nighttime; (2) a collaborative image processing method to detect and track the region of interest (ROI) as well as register images; and (3) a novel signal extraction method in time domain to accurately determine BR and HR.

## Methods

### Conflict of interests and ethics statement

This study was approved by the institutional review board (IRB) of the Shanghai Jiao Tong University and all participants provided full written informed consent. The individual in this manuscript has given written informed consent (as outlined in PLOS consent form) to publish these case details.

### Principle

**Fig 1:**
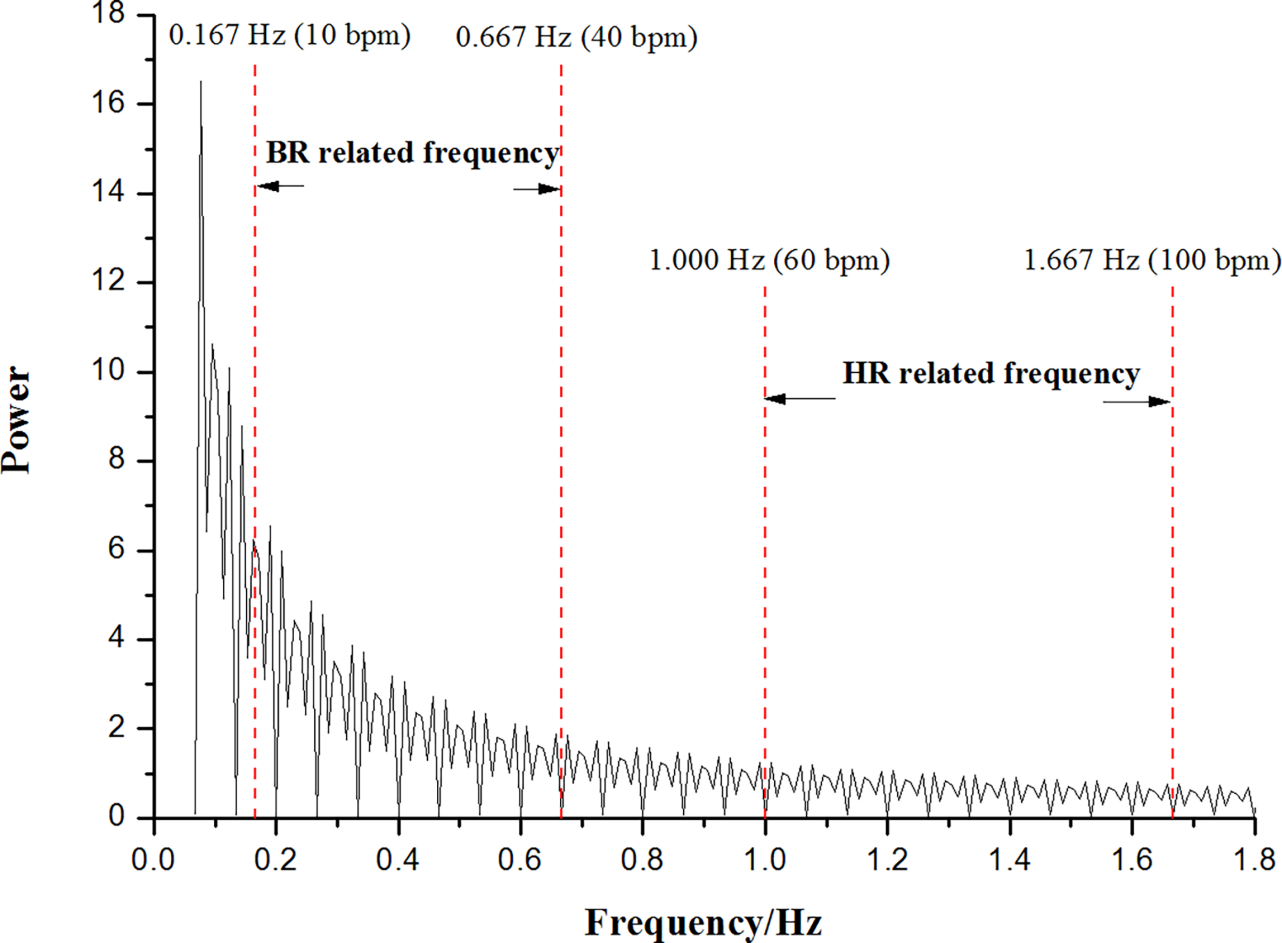
Illustration of BR and HR related frequencies on Fourier power spectrum extracted from one raw signal.

The disadvantage compared to the infrared image is that, due to few geometric and textural facial details, the thermal image is at present inadequate to design fast and reliable face detection algorithms [17]. Therefore, in the current work, the infrared image is used to assist in automatically recognizing face and facial tissue in thermal image. For BR, the principle of thermal imager is based on the fact that the temperature around the nose and mouth fluctuates throughout the inspiration and expiration cycle. In terms of HR, we can catch the subtle color variations caused by blood circulation from thermal video. However, these two targeted signals are mixed together in the raw extracted signal. Luckily, the common BR and HR are between 10–40 bpm and 60–100 bpm, respectively [18, 19]. As shown in **Fig 1**, there is no overlap for these two vital physiological indicators in the Fourier power spectrum, thus enabling the BR and HR to be measured simultaneously.

### A dual-camera imaging system

**Fig 2:**
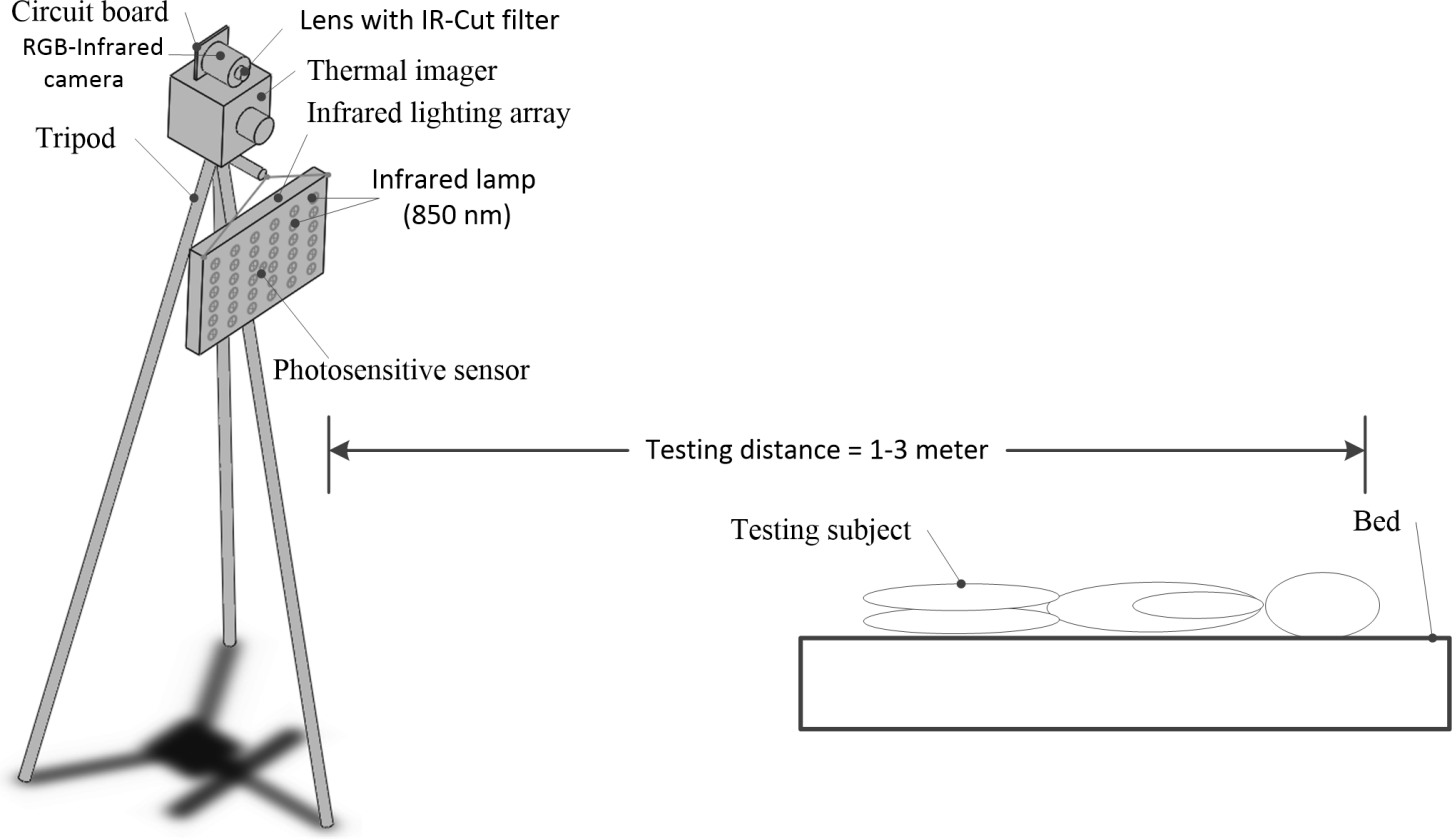
Experimental setup of this study.

**Fig 2**demonstrates the experimental setup. A thermal imager (MAG62, Magnity Electronics Co.Ltd., Shanghai, PR China) with the resolution of 640×480 and the pixel pitch of 17 μm is fixed on a tripod to prevent vibration during the experiments. Its spectral range and thermal sensitivity are 7.5–14 μm and 0.5°C, respectively. An infrared camera with the resolution of 640×480 is stabilized on the top of the thermal imager. These two cameras are parallel to each other in such a way that the field of view is almost the same. A USB and patch cables are applied for infrared and thermal cameras to connect a computer, respectively. A custom-built image acquisition software is developed for the generation of two trigger signals, thereby allowing the simultaneous acquisition of thermal and infrared videos. The obtained videos are afterwards loaded into the Matlab R2014a (The Mathworks, Inc., Natick, MA, USA) for further analysis.

**Fig 3:**
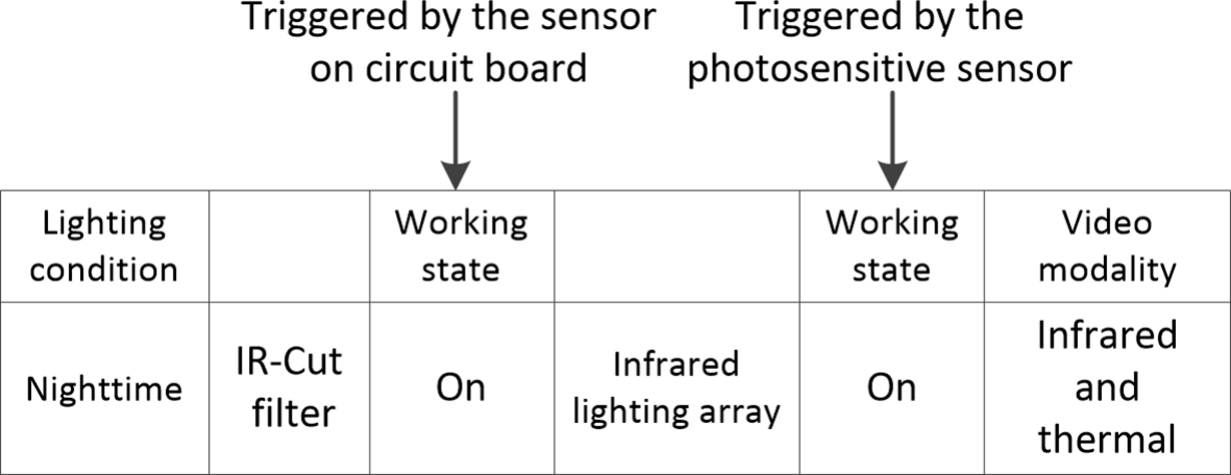
Workflow of the dual-camera system.

In order to record videos at night using the dual-camera system, a lens with IR-Cut filter is attached to the infrared camera, and this filter is triggered by sensor on the circuit board. In addition, an infrared lighting array having 36 infrared lamps is arranged below the imaging unit. The infrared lamps are arrayed in 6×6 matrix to realize the uniform illumination at night. A photosensitive sensor is installed in the middle of the infrared lighting array to control the working states of infrared lamps. The workflow of this dual-camera imaging system is elaborated in **Fig 3**to obtain the near-infrared and thermal videos during nighttime.

### A collaborative image processing method

During the nighttime, the infrared lamps begin working (**Fig 3**). For the purpose of accurately measuring the BR and HR under the no or low lighting situations, we exploit the respective advantages of infrared and thermal modalities. The thermal image is first used as reference to register infrared image. Subsequently, we conduct cross-modality data processing to detect and track the ROI. The raw signal containing the wanted BR and HR data is then extracted. The following sub-sections will describe these in detail.

#### Image registration

The affine transformation [20] is applied for registering thermal and infrared images. The control point pairs, viz., the moving points in the infrared image and the fixed points in the thermal image, are defined by manually choosing the strongly correlated points from the first frame of bimodal videos. The Eq (1) is used to do this

$$I_{vis\_r}=I_{vis}\;T,\;where\;T=\left[\begin{array}{ccc} scos\theta & ssin\theta & 0\\ -ssin\theta & scos\theta & 0\\ b_{x} & b_{y} & 1 \end{array}\right]$$ (1)

where I_vis_ is the original infrared image and its corresponding transformed infrared image is I_vis_r_; ***T*** represents the transformation matrix; ***s***, ***θ*** and ***b*** denote the scaling, rotation and translation vectors, respectively.

If the system keeps fixed during the experiment, there is no need to register it again.

#### Cross-modality ROI detection and tracking

**Fig 4:**
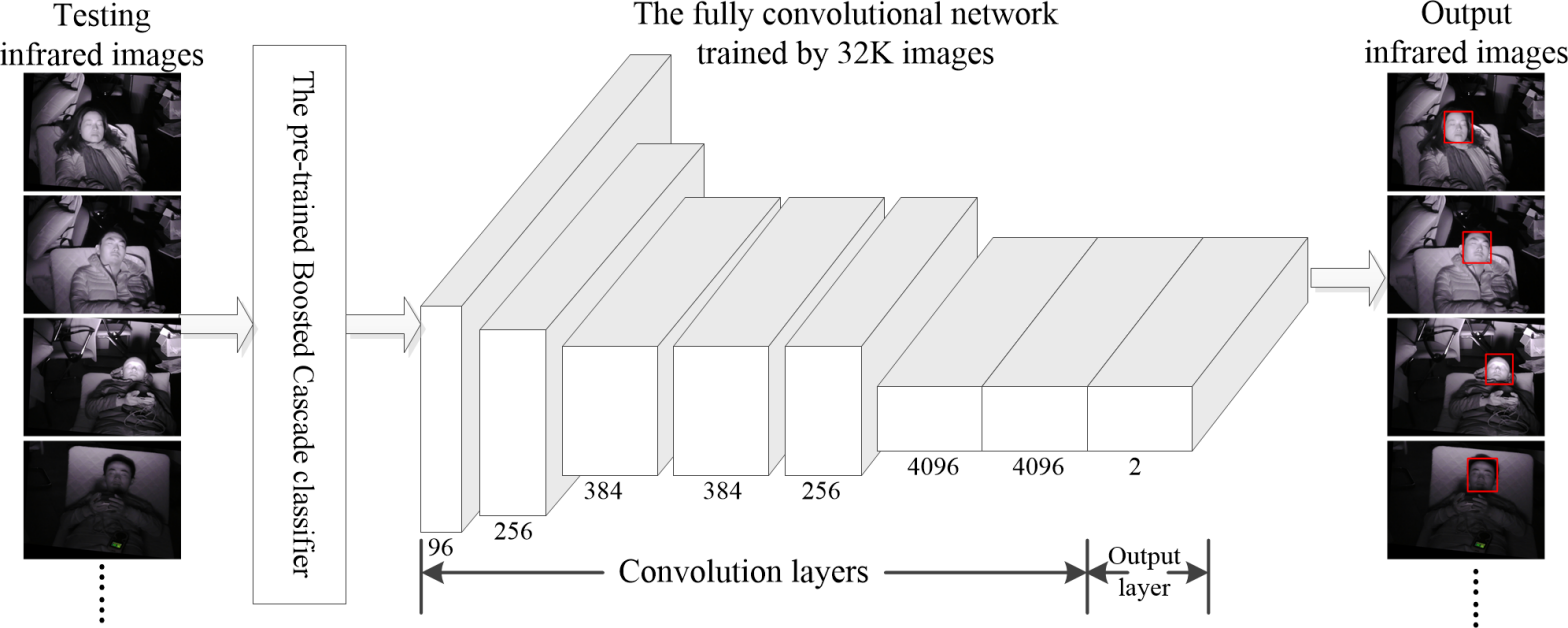
Near-infrared face detection via the combined use of the pre-trained Boosted Cascade classifier and deep classification net.

The first infrared image from the registered infrared video is imported into the pre-trained Boosted Cascade classifier [21] for locating the face region. When the pre-trained classifier fail to seek out the face, we exert the fully convolutional network [22], one of the classification nets for deep learning, to accomplish this task (**Fig 4**). To train the deep classification network, a total of 16,000 positive images (face) and the same number of negative images (background) are randomly selected from the PASCAL dataset [23] as the inputs of network. The detailed architecture of fully convolutional network used in this work can be found in **Fig 4**. By this means, almost any faces with different postures (e.g. side face) can be found in the infrared images.

**Fig 5:**
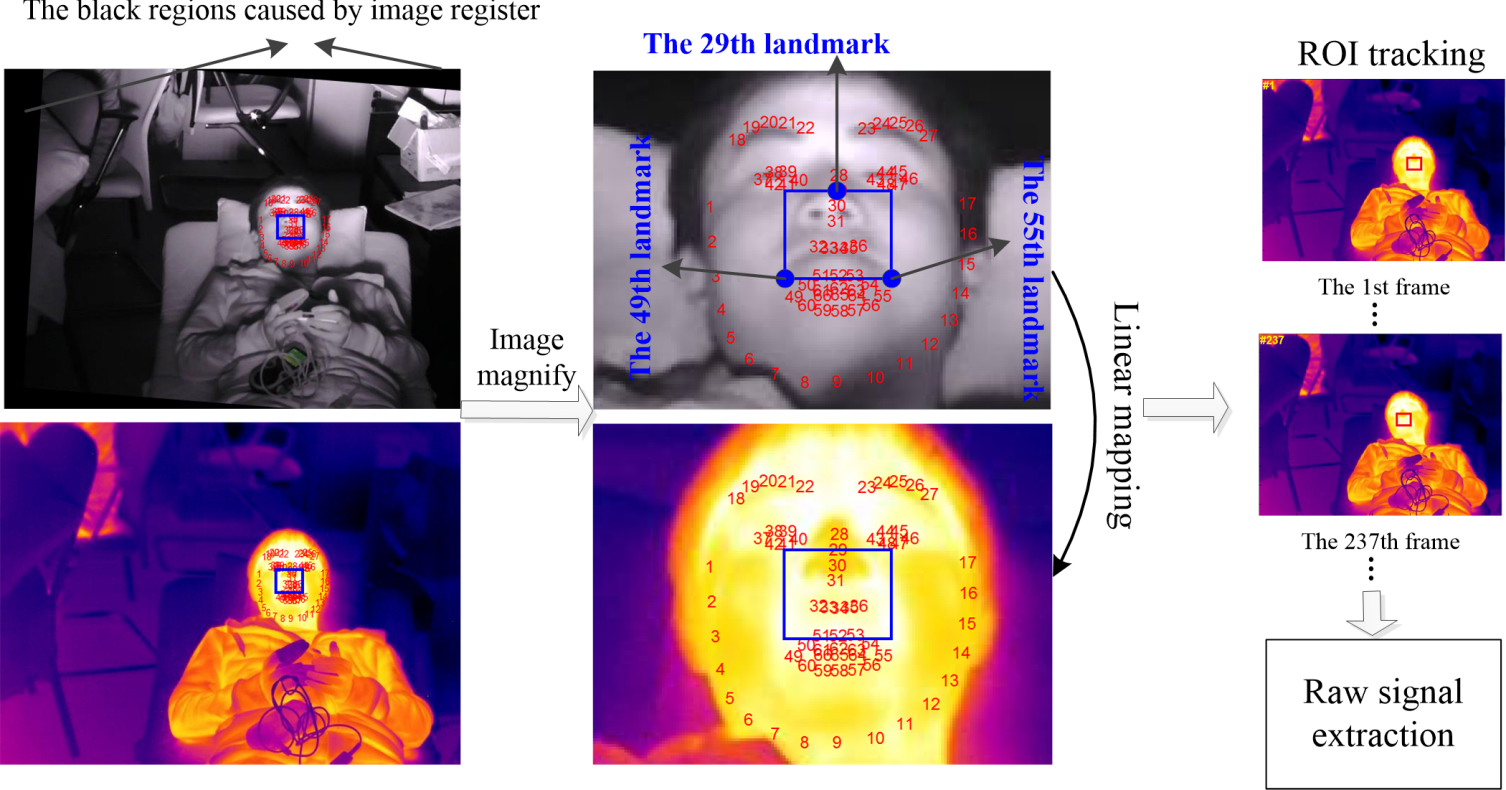
Cross-modality ROI detection and tracking.

After the face detection, a total of 66 landmark points are acquired by the discriminative regression based approach [24]. As shown in **Fig 5**, the 29th, the 49th and the 55th landmark points are selected to generate ROI in such a way that ROI can cover both the nose and mouth regions, thereby allowing us to measure BR no matter whether the subjects breathe using the nose and mouth simultaneously, alternately or individually. For HR measurement, the region between the nose and mouth contains the strongest heartbeat information. The ROI is calculated from these three points according to

$$\vec{ROI}=\left(\begin{array}{c} X\\ Y\\ H\\ W\\ \theta \end{array}\right)=\left(\begin{array}{c} y_{49}-H\cos\theta \\ x_{49}+H\cos\theta \\ \cos\theta {[y}_{29}-y_{49}-\tan\theta (x_{29}-x_{49})]\\ \left(x_{55}-x_{49}\right)/\cos\theta \\ \tan^{-1}\left(\left(y_{55}-y_{49}\right)/\left(x_{55}-x_{49}\right)\right) \end{array}\right)$$ (2)

where $\vec{ROI}$ is the ROI formation vector containing the horizontal ordinate (*X*), vertical coordinate (*Y*), height (*H*), width (*W*) and inclination angle (*θ*). The (*x*_29_,*y*_29_), (*x*_49_,*y*_49_) and (*x*_55_,*y*_55_) are the coordinates for the 29th, the 49th and the 55th landmarks, respectively.

Once the ROI region has been confirmed, the linear coordinate mapping is used to determine the corresponding region in thermal image. Subsequently, the off-the-shelf tracking algorithm called spatio-temporal context learning (STC) [25] is employed to track the ROI in the thermal image sequences (**Fig 5**). The average pixel intensity of ROI in thermal image is computed using the equation below

$$\bar{s}(k)=\frac{1}{n}\sum_{i,j\in N}s(i,j,k)$$ (3)

In equation, *s*(*i*,*j*,*k*) is the pixel intensity of thermal image at pixel (*i*, *j*) and video frame *k*; ***N*** is the vector of pixel coordinates in ROI and *n* is its number.

#### A signal extraction method in time domain

**Fig 6:**
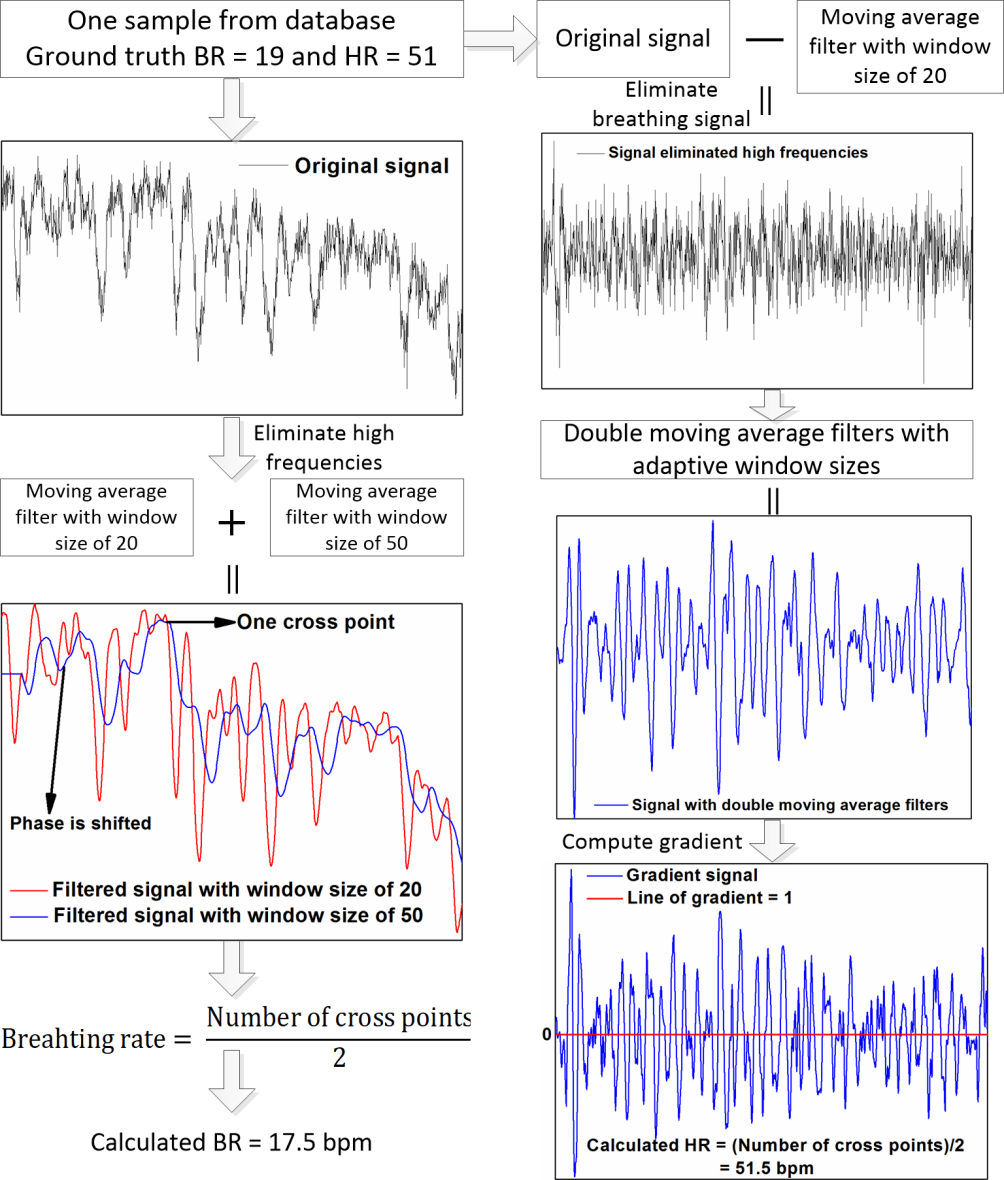
Proposed signal extraction method for BR (left column) and HR (right column) measurements.

Based on **Fig 1**, we can find that BR and HR signals are located on the low and high frequency areas of Fourier spectrum, respectively. Also, there exist some noises from the other factors such as the movement and illumination variation in the original signals. To accurately and robustly estimate BR and HR, we develop a time-domain signal extraction method (**Fig 6**). One sample are chosen from our database to clearly describe this method.

With respect to BR measurement, the high frequency parts mainly caused by the heartbeat and movement are first removed by moving average filter with window size of 20. The other curve is formed by phase shift, and a set of cross points yield. In our preliminary experiments, we observe that there is a hysteresis in the obtained signals when changing the window size of the sliding average window. This hysteresis is also called phase shift in this study (**Fig 6**). The larger window size, the more historical information in each processed data point. The hysteresis of signal using large window size is obviously larger than that using small window size. This generates the cross points between two processed signals. To eliminate the cross points brought by the overlap peaks of these two curves, the moving average filter with the larger window size viz., 50 is applied for the phase shifted curve. Subsequently, BR can be finally determined from the number of cross points (**Fig 6**, left column).

For HR measurement, the breathing-related data in original signal is first wiped off by subtracting the filtered signal with window size of 20. For the reason that the most noise events are belonged to high frequency parts, the two cascade moving average filters with adaptive window sizes are adopted to extract the relatively purified high frequencies. These adaptive window sizes vary from person to person. The differential signal is then derived with data interval of 3 to avoid the local extreme values. HR can be finally calculated from the cross points between the differential signal and line of gradient equal to 1 (**Fig 6**, right column).

Compared to the previous methods such as the Matlab function called findpeaks and fast Fourier transform, our approach can give more accurate and robust results (results not shown). The results shown in next section demonstrate that this approach is useful for BR and HR estimations from the image-derived signal.

#### A dual-mode sleep video database

A database of thermal and near-infrared dual-mode sleep videos is constructed to quantitatively verify the proposed dual-mode imaging system for BR and HR measurements under sleep situations. A total of 12 participants including different genders (two females and ten males) and ages (from 21 to 38 years old) consented to be subjects and were instructed to sleep with some body movements in multimedia lab of Shanghai Jiao Tong University. These participants were randomly recruited from Shanghai Jiao Tong University, School of Electronic Information and Electrical Engineering. Before the experiment, we asked the participant to close her/his eyes and rest in bed for ten minutes, and one dual-mode video was recorded while the participant was resting. Some volunteers were asked to be tested several times, and this database therefore had 28 video pairs crossing two domains. The testing distances were set from 1 to 3 meter (**Fig 2**). To simulate the sleep situation, the illumination intensity of the testing room was controlled within 3 Lux, which was monitored by the digital light meter (TA8131, TASI ELECTRONIC CO.,LTD., Jiangsu, PR China). During the video collection, the reference BR and HR were recorded by the dedicated and qualified human observers and ECG Monitor (PC-80B, Shenzhen Creative Industry Co.,Ltd., Guangzhou, PR China), respectively. The effectiveness of our approach was checked by the use of Bland-Altman plot [26, 27] and linear correlation analysis.

## Results and discussion

To test the performance of BR and HR measurements using bimodal imaging system in tandem with proposed algorithms in unobtrusive and contactless manner, two statistical analysis methods namely linear correlation analysis and Bland-Altman plot were applied for validating the data from small-scale pilot experiment.

### BR measurement

**Fig 7:**
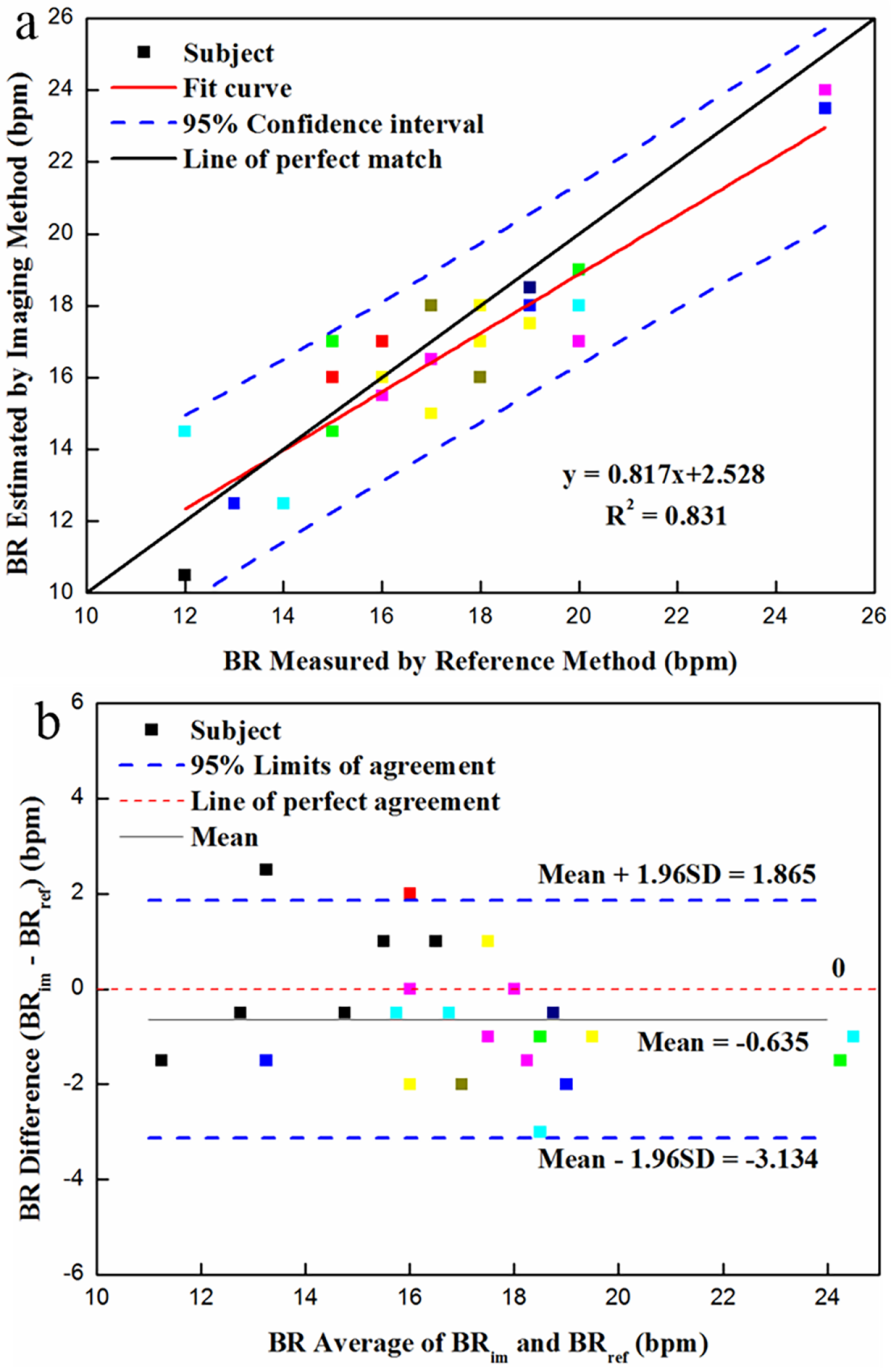
Statistical analysis of BR measurement using dual-mode imaging system under simulated sleep situation: (a) correlation between measured and reference BR; and (b) Bland-Altman plot (*N* = 26) of the difference against average for BR via the reference method (BR_ref_) and bimodal imaging system (BR_im_). (The different colors represent different volunteers).

The scatter plot and linear regression result of reference and measured BR are demonstrated in **Fig 7A**. As shown in **Fig 7A**, all scatter points located between the 95% upper and lower confidence intervals, and most of them were close to the line of perfect match, whose slope was equal to 1. From linear correlation analysis, the estimated BR was found to be relevant to the simultaneously-acquired reference BR with the determination coefficient (R^2^) of 0.831. The above results indicated that our approach was acceptable for BR measurement.

The corresponding Bland-Altman plot is shown in **Fig 7B** with the mean of difference of -0.635 bpm and limits of agreement of -3.134 and 1.865 bpm. The majority of scatter points were within the 95% limits of agreement, indicating that the measured BR was in good agreement with the reference BR. There was one black point out of upper limits of agreement with the offset approximately 0.635 bpm. The reason for this might be that the testing subject carried out one time very significant head motion during the experiment after we reviewed the original video. Several points were dispersed around the 95% limits of agreement, due in part to the highly low illumination (0~3 Lux) under simulated sleep situation. In conclusion, the result of Bland-Altman plot also revealed the capability of bimodal imaging system coupled with proposed algorithms for BR estimation during the night.

### HR measurement

**Fig 8:**
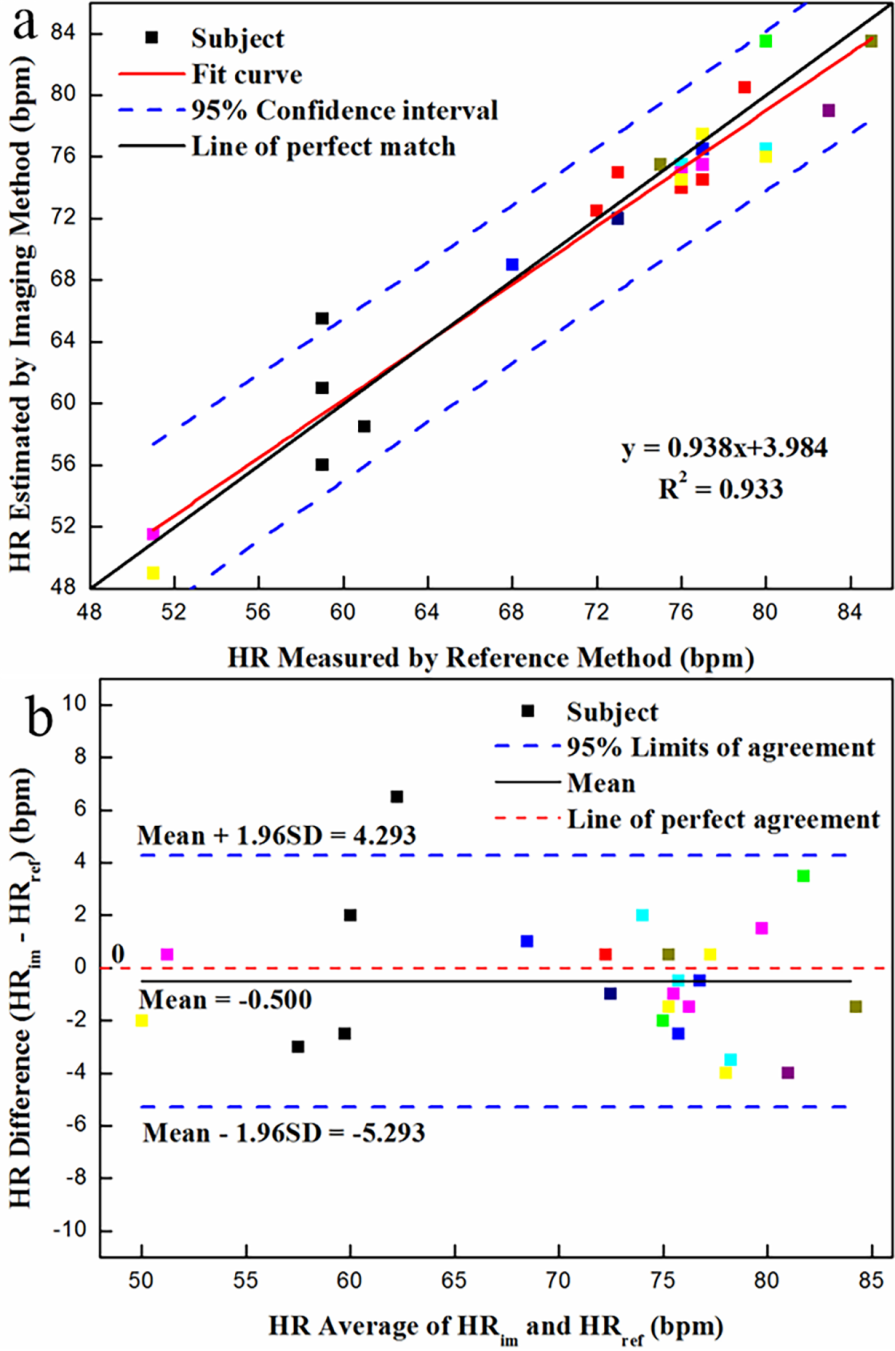
Statistical analysis of HR measurement using dual-mode imaging system under simulated sleep situation: (a) correlation between measured and reference HR; and (b) Bland-Altman plot (*N* = 25) of the difference against average for HR via the reference method (HR_ref_) and bimodal imaging system (HR_im_). (The different colors denote different volunteers).

In respect to HR measurement, the scatter plot and regression line are presented in **Fig 8A**. Apart from the one black point, the other testing data fell on the region between the two 95% confidence intervals. The strong correlation viz., R^2^ = 0.933 was observed between the measured and reference HRs, suggesting that the overall performance of the developed system is good for HR estimation. Although our system was to great extent robust against the low illumination, some unknown factors from experimental setup and environment would still influence the testing results. For example, by checking the original video, we cannot figure out the reason causing the black point out of the 95% confidence interval.

From the Bland-Altman plot shown in **Fig 8B**, in addition to one black point, all testing points located within the 95% upper and lower limits of agreement, which were 4.293 and -5.293 bpm, respectively. The distribution of points in **Fig 8B** was very similar with that of scatter points in **Fig 8A**. Consequently, the results of Bland-Altman analysis further supported these obtained by linear regression analysis.

## Discussion

Compared to the physiological signal measurement tools in the contact way during sleep [28,29], the contactless and unobtrusive optical approaches require more complex algorithms to analyze the obtained data. This is because the complicated imaging environments will increase the noise level of extracted signal and even deform the waveform. The most of published literature [30,31] used the depth camera to record the BR during sleep via the monitoring of the movement. However, the motion-based techniques such as active sonar [32] are sensitive to the significant body movement. Some investigators applied the RGB camera to estimate the BR and HR under the natural lighting conditions [14,33–35], but these system would not work when the subject slept during night. Hence, the state-of-art multi-camera system was developed for simultaneously measuring the BR and HR [15]. Despite the introduction of thermal camera, this system was infeasible for BR and HR measurement under the no or low lighting situations due to the 8×8 resolution of the thermal imagery. In contrast, we established the compact and effective dual-camera imaging system by adding the infrared camera equipped with IR-Cut filter and infrared lighting array (**Fig 2**), thus being able to measure BR and HR during nighttime.

## Conclusion

A dual-mode imaging system operating on short-wave infrared and long-wave infrared wavelengths associated with the proposed algorithm can synchronously measure BR and HR during nighttime. Using the data from the small pilot study under simulated sleep situation, the measured BR had relatively good relationship with the reference BR with determination coefficient of 0.831 via linear regression analysis. In terms of HR measurement, the measured HR was found to be strongly relevant to the reference HR with the determination coefficient of 0.933. The Bland-Altman plots of BR and HR exhibited the good performance of the dual-camera imaging system with limits of agreement of -3.134 and 1.865 bpm and -5.293 and 4.293 bpm, respectively. In general, the overall performance of the proposed technique is acceptable for BR and HR estimations during nighttime.
